# Correlation between newborn weight and serum BCAAs in pregnant women with diabetes

**DOI:** 10.1038/s41387-024-00301-6

**Published:** 2024-06-05

**Authors:** Na Tang, Yajin Liu, Sa Yang, Mengyu Zhong, Dongqing Cui, Ou Chai, Yurong Wang, Yunwei Liu, Xuejiao Zhang, Zhimin Hou, Haipeng Sun

**Affiliations:** 1 NHC Key Laboratory of Hormones and Development, Tianjin Key Laboratory of Metabolic Diseases, Chu Hsien-I Memorial Hospital & Tianjin Institute of Endocrinology, Tianjin Medical University, Tianjin, 300134 China; 2 Center for Cardiovascular Diseases, The Province and Ministry Co-Sponsored Collaborative Innovation Center for Medical Epigenetics, Tianjin Medical University, Tianjin, China

**Keywords:** Gestational diabetes, Nutrition, Proteins

## Abstract

**Background:**

Branched-chain amino acids (BCAAs), including leucine, isoleucine, and valine, are essential amino acids for mammals. Maternal BCAAs during pregnancy have been associated with newborn development. Meanwhile, BCAAs have been tightly linked with insulin resistance and diabetes in recent years. Diabetes in pregnancy is a common metabolic disorder. The current study aims to assess the circulating BCAA levels in pregnant women with diabetes and their relationship with neonatal development.

**Methods:**

The serum concentrations of BCAAs and their corresponding branched-chain α-keto acids (BCKAs) catabolites in 33 pregnant women with normal glucose tolerance, 16 pregnant women with type 2 diabetes before pregnancy (PDGM), and 15 pregnant women with gestational diabetes mellitus (GDM) were determined using a liquid chromatography system coupled to a mass spectrometer. The data were tested for normal distribution and homogeneity of variance before statistical analysis. Correlations were computed with the Pearson correlation coefficient.

**Results:**

The maternal serum BCAAs and BCKAs levels during late pregnancy were higher in women with PGDM than those in healthy women. Meanwhile, the circulating BCAAs and BCKAs showed no significant changes in women with GDM compared with those in healthy pregnant women. Furthermore, the circulating BCAA and BCKA levels in women with PGDM were positively correlated with the weight of the newborn. The circulating leucine level in women with GDM was positively correlated with the weight of the newborn. BCAA and BCKA levels in healthy pregnant women showed no correlation with newborn weight.

**Conclusions:**

The serum BCAAs in pregnant women with diabetes, which was elevated in PGDM but not GDM, were positively correlated with newborn weight. These findings highlight potential approaches for early identification of high-risk individuals and interventions to reduce the risk of adverse pregnancy outcomes.

## Introduction

Diabetes in pregnancy is a common metabolic disorder, which includes pregestational diabetes mellitus (PGDM) and gestational diabetes mellitus (GDM). There are about 200 million women with diabetes worldwide, 2/5 of whom are in their childbearing years. The prevalence of GDM is as high as 14.0% globally in 2021, with regional prevalence ranging from 7.1% to 27.6% [1]. Hyperglycemia during pregnancy has adverse effects on the mother and child, making them more susceptible to risks such as macrosomia and obstructed labor, and can also lead to adverse metabolic sequelae in the mother and offspring [2].

Branched-chain amino acids (BCAAs) are three essential amino acids consisting of leucine, isoleucine and valine. BCAAs, in addition to building proteins, play critical physiological roles in regulating cellular growth and metabolism [3]. Meanwhile, recent studies have shown that BCAAs are closely associated with a variety of pathological conditions such as metabolic diseases, cardiovascular diseases, and cancer [4–9]. BCAAs have attracted widespread attention as a biomarker of diabetes mellitus and a potential causal player in insulin resistance and diabetes [10–12]. BCAAs are strongly associated with insulin resistance and T2DM [13, 14]. In individuals with obesity, insulin resistance and diabetes mellitus, as well as in related animal models, plasma levels of BCAAs are significantly elevated [15–18]. The increased BCAAs is strongly associated with the high risk of future T2DM [10, 19]. Of note, most of the studies were performed in non-pregnant population.

Whether maternal circulating BCAA levels change in GDM remains controversial. It has been shown that, between GDM and non-GDM, there is no significant difference in the concentrations of circulating BCAAs [20]. There are also studies showing that blood concentrations of BCAAs are significantly lower in individuals with GDM compared to those with healthy pregnancies while leucine and isoleucine levels are associated with insulin resistance [21, 22]. On the other hand, it has been reported that circulating BCAA levels are elevated in individuals with GDM compared to those with normal glucose tolerance [23]. Further studies have shown that BCAA levels in early pregnancy in women with GDM are significantly different from those of controls even before the diagnosis of GDM [24, 25] and BCAAs can be predictive metabolites for GDM [26]. In PGDM, BCAA levels change during pregnancy. Women with PGDM have elevated BCAAs in their blood compared to healthy women [27]. In consistent with the increased BCAAs in non-pregnant women with type 2 diabetes, higher plasma concentrations of valine and isoleucine are detected in women with PGDM compared to those in healthy ones or ones with GDM [28].

BCAAs are essential nutrition for pregnancy. Previous studies have demonstrated a relationship between maternal BCAAs levels and fetal growth and development [29]. BCAAs in maternal urinary have also been found to be associated with intrauterine fetal growth and birth weight during pregnancy [30]. A large multiracial sample study shows that elevated maternal BCAAs during pregnancy is correlated to neonatal body size and/or insulin sensitivity and BCAAs are positively correlated with birth weight and/or number of skin folds [31, 32].

The current study aims to assess the circulating BCAA levels in women with GDM or PDGM and their relationship with neonatal development. The results showed that the serum levels of BCAAs and BCKAs during late pregnancy were elevated in women with PGDM but not GDM, compared with those of healthy women. There was no correlation between newborn weight and circulating BCAAs and BCKAs in healthy pregnant women. However, in women with PGDM and GDM, the weight of the newborn was positively correlated with circulating BCAA and BCKAs levels.

## Methods

This study was performed among pregnant women received antenatal care and gave birth in Chu Hsien-I Memorial Hospital & Tianjin Medical University between January to November in 2022. Sixty-four pregnant women aged 20–40 years were recruited and clinical data on the outcome of pregnancy were collected until delivery. Participants were divided into three groups: (1) 33 pregnant women with normal glucose tolerance, (2) 16 pregnant women with type 2 diabetes before pregnancy (PDGM), and (3) 15 pregnant women with GDM.

The inclusion criteria were as follows: gestational weeks at delivery ≥ 34 weeks and singleton pregnancy. The exclusion criteria were as follows: multiple pregnancies, stillbirth, in vitro fertilization-embryo transfer, type 1 diabetes, and chronic diseases requiring medication during pregnancy.

Blood samples from participants were venously collected after 8–14 h of fasting during 28–34 gestational weeks. Sample was centrifuged at 3500 g/min for 15 min at 4 °C and separation of serum were completed within 1 h. The serum samples were stored at −8 °C until retrieval for analysis.

This study was approved by the Ethics Review Committee of Chu Hsien-I Memorial Hospital of Tianjin Medical University and in accordance with the Helsinki Declaration. All study participants provided informed written consent prior to study enrollment.

The GDM cases were diagnosed by the oral glucose tolerance test (OGTT) conducted between 24 and 28 gestational weeks. Pregnant women were considered to have GDM if one of the following plasma glucose values was met or exceeded: 0 h, 5.1 mmol/L; 1 h, 10.0 mmol/L; or 2 h, 8.5 mmol/L, after a 75 g glucose load [33].

Using the Chinese reference charts [34], gestational age-adjusted standard deviation for birth weight was calculated. Small for gestational age (SGA) and large for gestational age (LGA) infants were defined as gestational age adjusted birth weights less than the 10th percentile and greater than the 90th percentile, respectively.

Body mass index (BMI)= body mass (kg)/metre squared of height (m^2^).

BCAA and BCKA levels in serum were determined using a Shimadzu LC-20AD liquid chromatography (LC) system coupled to an API 3200 electrospray-ionization triple-quadrupole mass spectrometer (AB SCIEX, Framingham, MA). The plasma levels of BCAA and BCKA were detected by multiple reaction monitoring (MRM) in positive and negative electrospray ionization mode, respectively. Chromatographic separation was achieved on an Agilent ZORBAX SB-C18 (150 × 3 mm, 5 μm) column, and temperature controlled at 50 °C. The standards and samples were separated using a mobile phase consisting of methanol/water (20:80, v/v) with 0.1% formic acid (eluent A) and acetonitrile (eluent B). The mobile was 0% B initially, which held for 1.5 min and then increased to 90% over 0.5 min. The mobile phase was held at 90% B for 5 min and then reequilibrated to 0% B and held for 6 min. The flow rate was 0.9 mL/min. An aliquot of 10 μL plasma was spiked with 110 μL methanol/acetonitrile/water (50:50:10, v/v/v) containing stable-isotope-labeled internal standards (48 ng [D3] Leucine, 24 ng [13C4, D3] KIV sodium salt, and 16 ng [D3] KIC sodium salt) and remained on ice for 10 min before being centrifuged at 14,000 g at 4 °C for 10 min. The supernatant was collected and dried with a stream of nitrogen. Following this, the samples were reconstituted in 100 μL of methanol/water (20:80, v/v) for analysis. The injection volume was 10 μL. Data acquisition and quantitation were performed with Analyst 1.7 and MultiQuant 3.0 software, respectively.

SPSS (V24.0, IBM Corp, Chicago, USA) was used to analyze data. The data were tested for normal distribution and homogeneity of variance before statistical analysis. Data were calculated as the mean ± standard. Categorical variable were reported as number and percentages. Differences between the three groups were tested by one-way analysis of variance (ANOVA), and Chi-square test was used for categorical data. Correlations were computed with the Pearson correlation coefficient. All tests were two-tailed, and *p* < 0.05 was considered statistically significant.

## Results

### Clinical characteristics of pregnant women included in the study

A total of 64 individuals, 33 normal pregnant women, 16 pregnant women with PGDM, and 15 pregnant women with GDM were included in this study. The clinical characteristics of the study participants were shown in Table 1.

**Table 1:** Clinical characteristics of pregnant women in PGDM, GDM, and control groups.

	Control (*n* = 33)	PGDM (*n* = 16)	GDM (*n* = 15)	F	*P*
Age (years)	31.52 ± 3.99	30.19 ± 4.00	34.13 ± 5.21#	3.404	**0.04**
Height (cm)	163.18 ± 4.54	162.69 ± 5.30	161.53 ± 5.01	0.597	0.554
Pre-pregnancy weight (kg)	63.80 ± 11.93	79.28 ± 15.70*	63.43 ± 12.15#	8.633	< 0.001
Pre-pregnancy BMI(kg/m2)	23.88 ± 4.03	29.77 ± 5.22*	24.22 ± 4.24#	10.386	< 0.001
Assisted reproduction technology(N)	5(15.15%)	1(6.25%)	1(6.67%)	0.942	0.66
Family history of diabetes(N)	2(6.06%)	7(43.75%)*	4(26.67%)	9.895	**0.006**
Family history of hypertension(N)	2(6.06%)	5(31.25%)	5(33.33%)*	7.592	**0.018**
Poor pregnancy history(N)	3(9.09%)	5(31.25%)	5(33.33%)	5.573	0.072
Gestational weight gain(kg)	15.18 ± 4.70	11.91 ± 5.36*	11.73 ± 3.68*	4.163	**0.02**
FPG(mmol/L)	4.66 ± 0.35	5.71 ± 1.04*	4.92 ± 0.63#	8.122	**0.002**
Complicated hypertension(N)	3(9.09%)	8(50.00%)*	4(26.67%)	9.776	**0.005**

^*^means compared with the control group, *p* < 0.05;

# means compared with the PGDM group, *p* < 0.05;

The bold values denote statistical significance at *p* < 0.05 level.

No significant differences were observed on the height, assisted reproduction technology, or poor pregnancy history among women with PGDM or GDM and controls. Meanwhile there were significant differences in age, pre-pregnancy weight, pre-pregnancy BMI, family history of diabetes, family history of hypertension, weight gain during pregnancy, fasting plasma glucose (FPG), and complicated hypertension among the three groups (*p* < 0.05). Notably, women with PGDM had the highest pre-pregnancy weight, pre-pregnancy BMI, family history of diabetes mellitus, fasting plasma glucose, and complicated hypertension during pregnancy. The women in control group had the highest weight gain during pregnancy (*p* < 0.05).

### Characteristics of the newborns

The information of the newborns was collected after delivery by women included in the study and the clinical characteristics of the newborns were shown in Table 2. There were no significant differences in the weight and sex of newborns among the three groups. There were significant differences in gestational weeks of delivery and neonatal weight distribution among the three groups (*p* < 0.05). The shortest gestational weeks of delivery and the highest proportion of LGA were revealed in PGDM group. The proportion of newborn with appropriate age delivered in PGDM and GDM groups was significantly lower than that of control group.

**Table 2:** Characteristics of the newborns delivered by women in PGDM, GDM, and control groups.

		Control (*n* = 33)	PGDM (*n* = 16)	GDM (*n* = 15)	F/X^2^	*P*
Gestational weeks of delivery (weeks)		38.42 ± 1.20	37.50 ± 1.32^*^	37.93 ± 1.10	3.295	**0.044**
Newborn weight (g)		3326.06 ±436.11	3627.19 ± 780.24	3287.33 ± 578.14	1.118	0.342
Neonatal sex	Male	14(42.42%)	10(62.50%)	10(66.67%)	3.187	0.203
	Female	19(57.58%)	6(37.50%)	5(33.33%)		
Newborns	SGA	2(6.06%)	3(18.75%)	4(26.67%)		
	Appropriate gestational age	27(81.82%)	5(31.25%)^*^	7(46.66%)^*^	14.561	**0.003**
	LGA	4(12.12%)	8(50.00%)^*^	4(26.67%)		

^*^means compared with the control group, *p* < 0.05;

The bold values denote statistical significance at *p* < 0.05 level.

### Serum concentrations of BCAAs and BCKAs in pregnant women

**Fig. 1:**
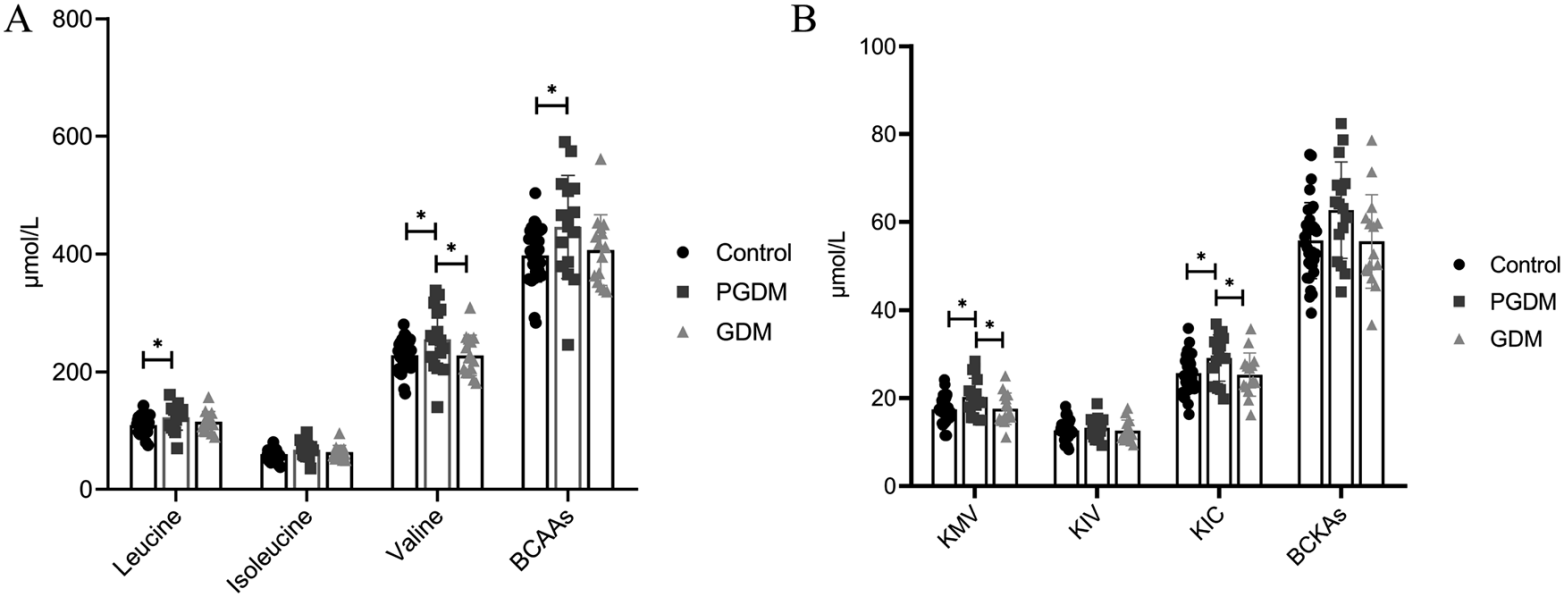
Serum levels of BCAAs and BCKAs in control, PGDM, and GDM group. **A** Leucine, isoleucine, valine and BCAAs concentrations in the serum of the different groups. **B** KIC, KMV, KIV and BCKAs concentrations in the serum of the different groups. KIC, α-ketoisocaproate; KMV, α-keto-β-methylvalerate; KIV, α-ketoisovalerate. *, compared with the control group, *p* < 0.05; #, compared with the PGDM group, *p* < 0.05.

We employed LC-MS for the quantification of serum levels of BCAAs and the corresponding BCKAs including α-ketoisocaproate (KIC, ketoleucine), α-keto-β-methylvalerate (KMV, ketoisoleucine), and α-ketoisovalerate (KIV, ketovaline). The serum levels of BCAAs and BCKAs across the three groups are graphically depicted in Fig. 1, with detailed data presented in Table 3. There was no significantly difference in the serum levels of BCAAs and BCKAs between GDM and control groups. Notably, the serum levels of BCAAs were significantly elevated in the PGDM group (445.78 ± 87.63 μmol/L) in comparison to the control group (397.52 ± 45.27 μmol/L). leucine (122.76 ± 22.04 μmol/L) and valine (255.48 ± 53.58 μmol/L) were significantly elevated, compared with those in the control group (109.50 ± 14.21 μmol/L, 228.08 ± 26.01 μmol/L). The serum levels of KIC and KMV were significantly higher in the PGDM group (29.16 ± 5.24 μmol/L, 20.26 ± 4.32 μmol/L) compared with those in the control group (25.69 ± 4.64 μmol/L, 17.44 ± 2.90 μmol/L) and GDM group (25.35 ± 4.93 μmol/L, 17.60 ± 3.58 μmol/L).

**Table 3:** The serum levels of BCAAs and BCKAs in PGDM, GDM, and the control groups.

(μmol/L)	Control (*n* = 33)	PGDM (*n* = 16)	GDM (*n* = 15)	F	*P*
BCAAs	397.52 ± 45.27	445.78 ± 87.63^*^	406.50 ± 60.26	3.375	**0.041**
Leucine	109.50 ± 14.21	122.76 ± 22.04^*^	115.18 ± 17.23	3.261	**0.045**
Isoleucine	59.94 ± 8.63	67.54 ± 14.74	63.40 ± 11.27	2.607	0.082
Valine	228.08 ± 26.01	255.48 ± 53.58^*^	227.93 ± 34.49^#^	3.39	**0.04**
BCKAs	55.79 ± 8.64	62.71 ± 11.00	55.57 ± 10.65	3.085	0.053
KIC	25.69 ± 4.64	29.16 ± 5.24^*^	25.35 ± 4.93^#^	3.269	**0.045**
KMV	17.44 ± 2.90	20.26 ± 4.32^*^	17.60 ± 3.58^#^	3.875	**0.026**
KIV	12.66 ± 2.15	13.29 ± 2.39	12.63 ± 2.38	0.481	0.621

*means compared with the control group, *p* < 0.05;

# means compared with the PGDM group, *p* < 0.05;

The bold values denote statistical significance at *p* < 0.05 level.

### Correlation between newborn weight and serum levels of BCAAs and BCKAs in healthy pregnant women

**Fig. 2:**
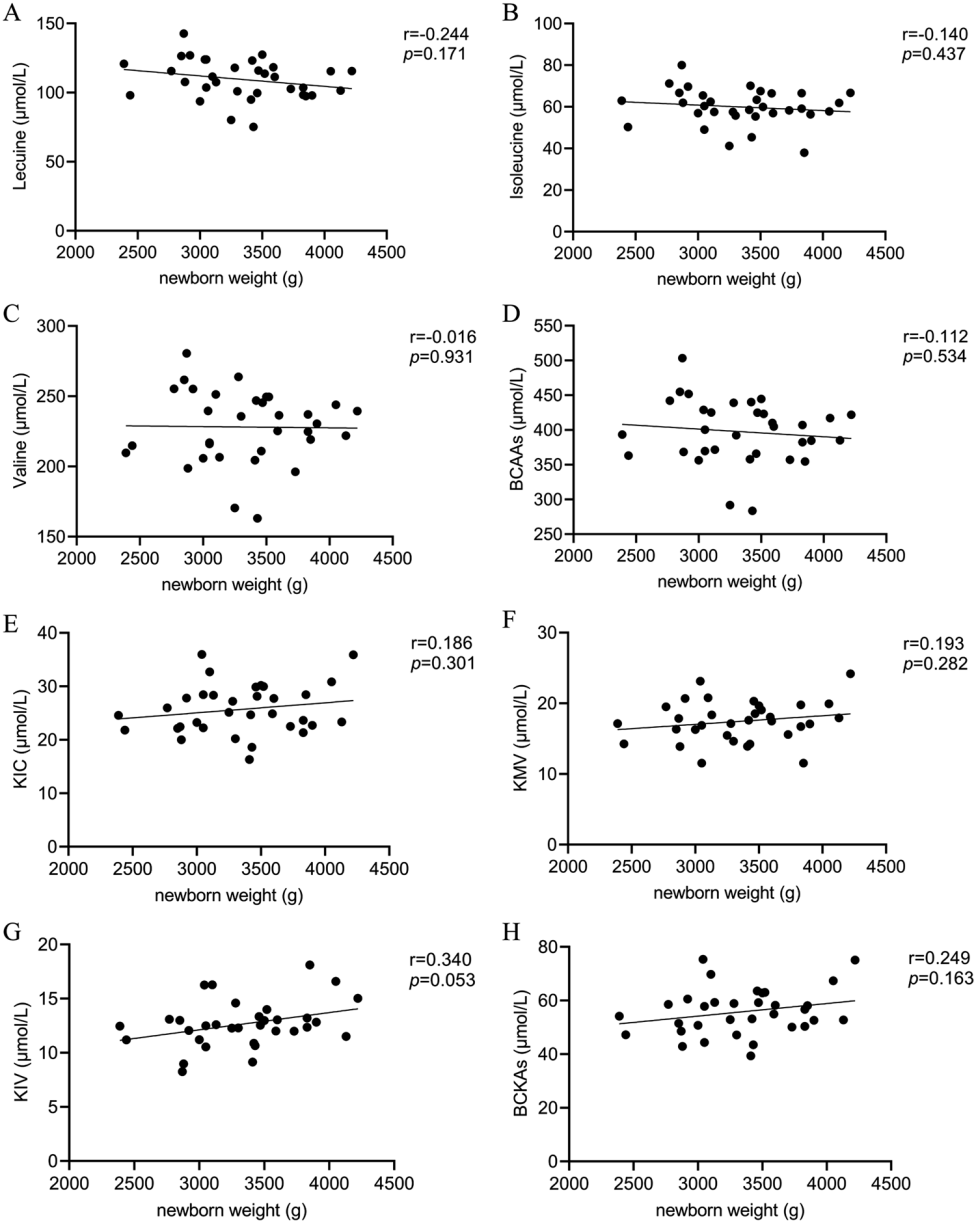
Correlation analysis between newborn weight and serum BCAA and BCKA levels in healthy pregnant women. **A**–**H** The correlation between newborn weight and serum level of leucine, isoleucine, valine, BCAAs, KIC, KMV, KIV, and BCKAs, respectively. “r” represents the correlation coefficient. *p* < 0.05 is considered statistically different.

Correlation analysis was performed to examine the relationship between serum BCAA and BCKA levels and newborn weight among healthy pregnant women. The results showed no significant correlation between newborn weight and BCAA or BCKA levels (*p* > 0.05), as shown in Fig. 2.

### Correlation between neonatal weight and serum BCAAs and BCKAs in PGDM group

**Fig. 3:**
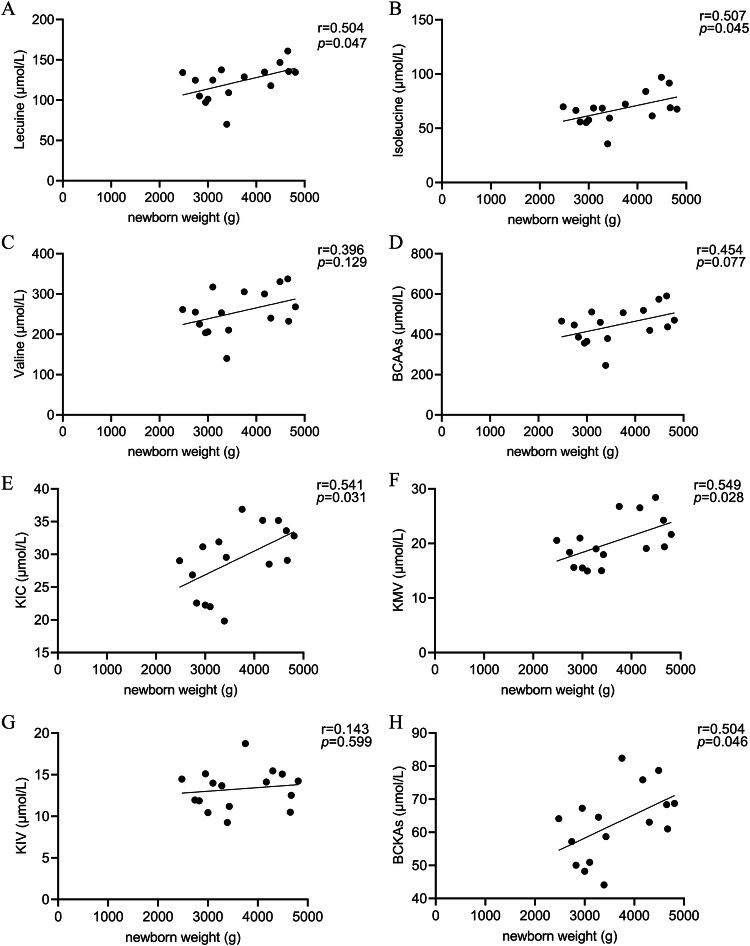
Correlation analysis between newborn weight and serum BCAA and BCKA levels in PGDM group. **A**–**H** The correlation between newborn weight and serum level of leucine, isoleucine, valine, BCAAs, KIC, KMV, KIV, and BCKAs, respectively. “r” represents the correlation coefficient. *p* < 0.05 is considered statistically different.

We further analyzed the correlation between newborn weight and serum BCAA and BCKA levels in women with PDGM. The correlation analysis revealed significant associations. The serum levels of leucine, isoleucine, KIC, KMV, and BCKAs were positively correlated with newborn weight in PGDM group (r = 0.504 *p* = 0.047; r = 0.507 *p* = 0.045; r = 0.541 *p* = 0.031; r = 0.549 *p* = 0.028; r = 0.504 *p* = 0.046, respectively). The results were represented in Fig. 3.

### Correlation between newborn weight and serum BCAAs and BCKAs in GDM group

**Fig. 4:**
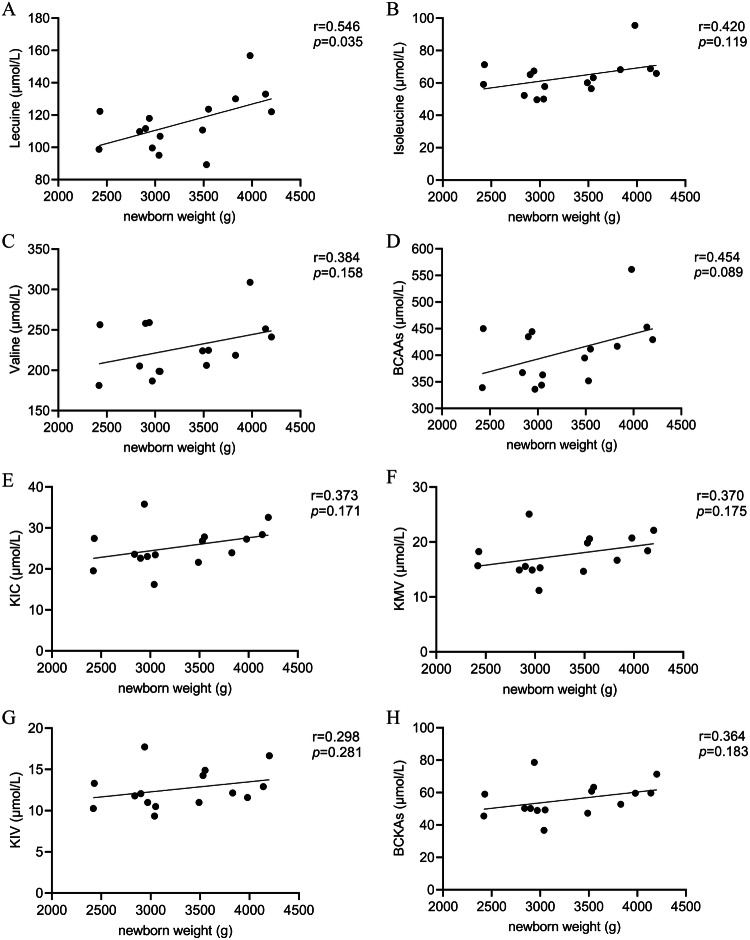
Correlation analysis between newborn weight and serum BCAA and BCKA levels in GDM group. **A**–**H** The correlation between newborn weight and serum level of leucine, isoleucine, valine, BCAAs, KIC, KMV, KIV, and BCKAs, respectively. “r” represents the correlation coefficient. *p* < 0.05 is considered statistically different.

Further correlation analysis revealed significant associations between newborn weight and serum BCAA levels in pregnant women with GDM. There was a positive correlation between circulating leucine level with newborn weight (r = 0.546 *p* = 0.035). No significant correlation between circulating BCKAs with newborn weight was observed. *p* < 0.05 is considered statistically different. The results were represented in Fig. 4.

## Discussion

The current study showed that the serum BCAAs and BCKAs levels were elevated in women with PGDM but not GDM. Of note, in both groups but not healthy pregnant women, the circulating BCAA levels were positively correlated with the weight of the newborn.

Our data showed that circulating BCAA levels in pregnant women with PGDM were significantly higher than those in healthy pregnant women, which is consistent with findings in non-pregnant individuals with diabetes [35] and women with PGDM [28]. The serum BCAA levels in late pregnancy in GDM showed a trend of increase without statistical significance, compared to those in healthy women. One previous study found no significant change in BCAA levels in in early pregnancy in GDM [20]. Another study at late pregnancy found BCAAs were increased in women with GDM [23]. More studies with big cohorts will help to better determine the BCAA changes in GDM.

Our data showed that, either in PGDM or GDM, the serum levels of BCAAs in mothers were positively correlated with newborn weight. Of note, the BCAA levels in GDM was not elevated. It remains unclear how the positive correlation is establishment. Our data indicated that neonatal weight was not affected by mothers’ body weight and gestational weeks of delivery. We found that, while there was no statistically significant difference in average neonatal weight among 3 groups, the distribution of neonatal weight was significantly different with the highest proportion of LGA in PGDM group. The positive correlation between maternal BCAAs and newborn weight suggests a potential predictor of neonatal obesity in diabetic pregnancy.

We found that the levels of BCAAs in pregnant women with normal glucose tolerance do not correlate with the weight of the newborns. However, previous studies on maternal BCAAs and fetal growth in healthy pregnancies found that BCAAs are positively correlated with neonatal birth weight [31, 32]. Discrepancy could be attributed to differences in ethnic origin, age, living and dietary habits, sampling time, or the size of cohort.

The current study has its limitations. The sample size of this study was relatively small and future studies with larger cohorts are needed. In addition, we analyzed serum BCAAs only in the late stages but not the early and middle trimesters of pregnancy. Given the potential metabolic changes among trimesters, it is of interest to analyze BCAAs throughout pregnancy.

The changes of BCAAs and their correlation with newborn weight in pregnant women with diabetes provide insight into the crosstalk between maternal metabolism and newborn size. These findings highlight the importance of evaluating the roles of protein nutrient and amino acid metabolism in GDM and PDGM, and suggest potential strategies for determining risk factor and reducing the risk of adverse pregnancy outcomes.

## Data Availability

All data generated or analyzed during this study are included in this published article.
